# Exploring public opinion on health effects of prepared dishes in China through social media comments

**DOI:** 10.3389/fpubh.2024.1424690

**Published:** 2024-09-12

**Authors:** Tao Shu, Han Yang, Ling Lin, Jian Chen, Jixian Zhou, Jun Wang

**Affiliations:** ^1^School of Software Engineering, Chengdu University of Information Technology, Chengdu, China; ^2^School of Computer Science, Chengdu University of Information Technology, Chengdu, China; ^3^School of Computing and Artificial Intelligence, Southwestern University of Finance and Economics, Chengdu, China; ^4^College of Blockchain Industry, Chengdu University of Information Technology, Chengdu, China; ^5^School of Management Science and Engineering, Southwestern University of Finance and Economics, Chengdu, China

**Keywords:** prepared dishes, public opinion, social media comments, topic modeling, sentiment analysis

## Abstract

**Introduction:**

In the 2020s, particularly following 2022, the Chinese government introduced a series of initiatives to foster the development of the prepared dishes sector, accompanied by substantial investments from industrial capital. Consequently, China’s prepared dishes industry has experienced rapid growth. Nevertheless, this swift expansion has elicited varied public opinions, particularly concerning the potential health effects of prepared dishes. Therefore, this study aims to gather and analyze comments from social media on prepared dishes using machine learning techniques. The objective is to ascertain the perspectives of the Chinese populace on the health implications of consuming prepared dishes.

**Methods:**

Social media comments, characterized by their broad distribution, objectivity, and timeliness, served as the primary data source for this study. Initially, the data underwent preprocessing to ensure its suitability for analysis. Subsequent steps in this study involved conducting sentiment analysis and employing the BERTopic model for topic clustering. These methods aimed to identify the principal concerns of the public regarding the impact of prepared dishes on health. The final phase of the study involved a comparative analysis of changes in public sentiment and thematic focus across different time frames. This approach provides a dynamic view of evolving public perceptions related to the health implications of prepared dishes.

**Results:**

This study analyzed over 600,000 comments gathered from various social media platforms from mid-July 2022 to the end of March 2024. Following data preprocessing, 200,993 comments were assessed for sentiment, revealing that more than 64% exhibited negative emotions. Subsequent topic clustering using the BERTopic model identified that 11 of the top 50 topics were related to public health concerns. These topics primarily scrutinized the safety of prepared dish production processes, raw materials, packaging materials, and additives. Moreover, significant public’s interest was in the right to informed consumption across different contexts. Notably, the most pronounced public opposition emerged regarding introducing prepared dishes into primary and secondary school canteens, with criticisms directed at the negligence of educational authorities and the ethics of manufacturers. Additionally, there were strong recommendations for media organizations to play a more active role in monitoring public opinion and for government agencies to enhance regulatory oversight.

**Conclusion:**

The findings of this study indicate that more than half of the Chinese public maintain a negative perception towards prepared dishes, particularly concerning about health implications. Chinese individuals display considerable sensitivity and intense reactions to news and events related to prepared dishes. Consequently, the study recommends that manufacturers directly address public psychological perceptions, proactively enhance production processes and service quality, and increase transparency in public communications to improve corporate image and people acceptance of prepared dishes. Additionally, supervisory and regulatory efforts must be intensified by media organizations and governmental bodies, fostering the healthy development of the prepared food industry in China.

## 1 Introduction

In recent years, the convenience food market has expanded rapidly due to the accelerated pace of life and the impact of the epidemic, driving demand for convenient food products (1, 2).The global convenience food market revenue may reach 653.6 billion US dollars in 2024, and most of the revenue, about 154 billion US dollars will come from China, accounting for approximately 23.56% of the total market (3). According to statistics, the market size of Chinese prepared dishes reached 48.54 billion US dollars in 2022, with an increase of 21.3%. China’s prepared dishes market is also expected to maintain a high growth rate in the future, and the scale of the prepared dishes market will reach 1,072 billion yuan in 2026 (4).

This growth has made prepared dishes a hot spot in both academic and industrial sectors. Existing studies have elucidated the definition that prepared dishes are a general term for foods that are convenient to cook, people do not need to cook or simply cook to eat (5, 6), which is finished or semi-finished dishes made with one or more agricultural products as the main raw materials and are pre-processed or pre-cooked and pre-packaged through standardized flow operations (7, 8). Furthermore, the classification, production, preservation and other technologies of prepared dishes were researched (9–15). Particularly in the context of China’s rapidly growing prepared dishes market, researchers have begun to focus on public opinion regarding these products. Various studies have revealed, through questionnaire surveys and in-depth interviews, the factors including perceived risk and trust (16), attitude, subjective norm, and perceived control (17), different consumer cognition and product features (18–20), affecting the consumption intention of prepared dishes. Also, market research reports have been driven by consumption events. For example, during the 2022 Chinese New Year, a survey indicated high purchase rates of prepared dishes (over 80%), with 60% choosing them for the Chinese New Year dinner. Despite widespread acceptance, over 60% of consumers expressed dissatisfaction, pointing to a gap between product offering and consumer expectation (21). The reason for such contradictory situations is closely related to Chinese society and food culture. In the view of most Chinese, dishes not only meet the survival requirements of human beings but also are closely related to people’s attitudes towards life and artistic aesthetics, so they highly value individuality and oppose standardization. Following Chinese family traditions, personally cooking for family members is also important to family ethics. For complex reasons, most Chinese prefer to rely on natural food and condiment raw materials and reject chemical synthetic materials. Most Chinese think additives are very unhealthy. At the same time, Chinese people excessively reject long-term preserved food for nutrition loss during the preservation process and advocate fresh ingredients. In addition, Chinese people pay more attention to their offspring’s health than their own. So Chinese consumers’ current high acceptance of prepared dishes is a helpless compromise in the face of social changes and life pressures. Thus, it is easy to understand why the recent introduction of prepared dishes on campus has raised concerns about nutritional adequacy, leading the majority of people to hold an opposing attitude, particularly parents worried about their children’s health (22). Subsequently, the dialogue around public health concerns about prepared dishes has become increasingly prominent. Thus, public perceptions regarding the health effects of prepared dishes have also become an imperative concern in China.

However, previous related work has not focused sufficiently on public opinion regarding the health effects of prepared dishes and still relies on traditional data collection methods such as closed-ended questionnaire surveys, which have limitations in terms of quantity, timeliness, duration, geographic scope, richness, and objectives (23, 24). While survey experiments play a vital role in understanding people perceptions, they might be influenced by biases such as social desirability bias, response bias, and common method bias (25, 26). Additionally, some psychometric questionnaires struggle to capture human emotions, which are subjectively experienced in specific contexts, making their objective assessment difficult (27, 28). Consequently, it becomes challenging to grasp prompt public opinion on health effects of prepared dishes and observe the constant change in attention. Some scholars argue that adoption of more objective data and research methods in the studies on food industry can help to resolve these problems (29, 30). Fortunately, with the rapid progress of information and communication technology, especially influential social media platforms, myriad user-generated contents (UGCs) are recorded, stored, and accumulated, forming an important type of big data, which are publicly available, easily collected, low cost, spontaneous, passionate and insightful (31). According to the report, the number of people using social media worldwide has reached 4.74 billion in 2022 and is expected to reach 5.85 billion by 2027, affecting more than half of the world’s population (32). People find social media platforms convenient to share their opinions (33), sentiments (34), attitudes (35), purchase intention (36), etc., through comments by natural language. Social media comments are notable not just for their depth and usability but also for the automated retrieval of this UGCs, representing a significant technological advancement in public psychological perception analysis (37, 38), and can serve as a valuable supplementary data source for research in food industries (39, 40). These dynamic data augments traditional survey methodologies by offering a deeper understanding, tracking evolving trends, temporal patterns, and spatiotemporal patterns across various scales—particularly beneficial in areas where gamut surveys are lacking (41, 42).

Recently, in the food domain, mining and analyzing user-generated comment text from social media platforms can provide us with valuable insights into public opinion analysis for health effects of prepared dishes (30, 40). Scholars could explore the public attitude towards organic foods through Twitter posts (29), natural food products opinion expressed on social media comments (43), “coffee’s effects on health” information about food product attributes perception sharing on Twitter (44) and public perceptions regarding alternative meat through Sina Weibo (45). Simultaneously, the application of natural language processing (NLP) methods—including text preprocessing, topic modeling, sentiment analysis, and machine learning or deep learning (24, 29, 30, 40, 44–47)—to food-related text data analysis enables scholars to extract valuable insights from comment corpus without the cumbersome computational work (48, 49). In contrast to conventional way to collect peoples’ responses, social media comments and NLP approaches could deliver us current public perception with big data dynamically and get real-time feedback which is very vital in this fast-changing world. Nonetheless, the Chinese public opinion on health effects of prepared dishes has not been fully studied, particularly using objective data from social media.

Therefore, compared to previous research, this study pivots towards a fresh perspective by harnessing the expansive reach and immediacy of social media platforms to capture and analyze public opinions on the health effects of prepared dishes in China. By focusing explicitly on mining public opinions from social media, this study not only taps into real-time public perception but also broadens the scope of analysis beyond the limitations of conventional data collection methods. It presents an opportunity to explore the multifaceted nature of public discourse surrounding the health implications of prepared dishes, capturing a broad spectrum of opinions. This approach enables a timely and detailed examination of public’s concerns, preferences, and expectations, providing valuable insights that could influence policy-making, inform industry strategies, and encourage the development of healthier convenience food options.

The remainder of the paper is structured to unfold as follows: Section 2 introduces the methodology employed for collecting and analyzing social media comments to gauge public opinion on the health effects of prepared dishes in China. Section 3 discusses the findings, shedding light on the public’s health concerns. Section 4 concludes with a summary of key insights, offering implications for the prepared dishes industry and public health strategies. Section 5 analyzes the study’s limitations, introducing future research directions to understand public opinion and navigate the complexities of public health perceptions.

## 2 Research design and methods

### 2.1 Research design

This study introduces an analytical framework for big data, emphasizing the importance of examining all comments on social media platforms to capture a comprehensive public opinion on the health effects of prepared dishes in China. Theoretical foundations suggest that including diverse perspectives, regardless of individual prominence, allows for a more representative aggregation of opinions (29, 41, 45, 50). To facilitate this extensive analysis, the study uses a set of NLP techniques to increase automation efficiency. Connecting theory to practice, data analysis in this context is carried out using two distinct methodologies: top-down and bottom-up (51, 52). In a top-down approach, the process starts with identifying a specific problem and using domain-specific knowledge to form hypotheses, which are later tested through data analysis. In contrast, this study uses a bottom-up approach, avoiding predetermined hypotheses and instead focusing on thorough data collection and analysis. This method depends on using algorithms to discover hidden patterns, providing insights directly drawn from the data.

**Figure 1:**
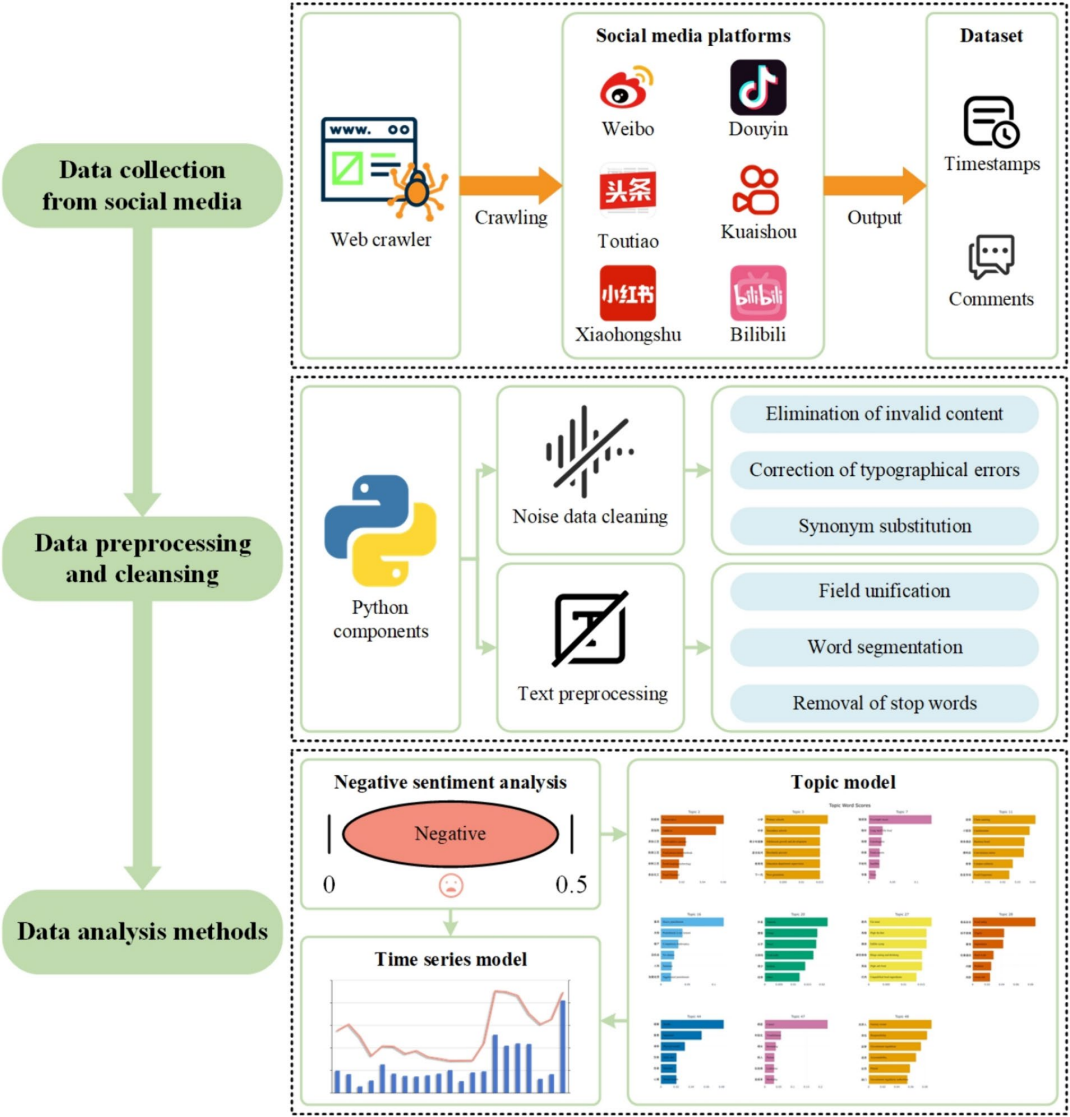
Research framework.

Continuing from the methodological foundations laid out, the study’s focus shifts to the application of these principles in understanding the opinions of the Chinese public towards health effects of prepared dishes. By not presupposing any initial hypotheses, the research remains open to discovering authentic public opinions, irrespective of the data’s origin. This method is especially useful for examining sentiment trends and health topics in negative comments as they change over time. The transition from theoretical underpinning to practical application informs the subsequent steps in the research process. After establishing the methodology, the selection of social media comments for analysis was guided by the objective to capture a wide range of opinions. This objective consideration shapes the overall research design, which is detailed in the subsequent sections covering data collection, preprocessing, and analytical techniques. Each phase of the workflow is interconnected, ensuring that the study’s design and execution are coherent and aligned with the overarching goal of understanding publics’ opinion about the health effects of prepared dishes in a comprehensive and unbiased manner. Based on the above thoughts and methods, the research framework is depicted in Figure 1, outlining the methodological steps as follows:

Step 1: data collection from social media. Following the research goals, the initial step involves selecting social media platforms (e.g., Weibo, Toutiao, which are Twitter-like social media platform in China) based on their influence and data availability (53, 54). This stage includes determining the types of data to be collected and the methods for data collection.

Step 2: data cleansing and preprocessing. This crucial step enhances data quality and reliability by eliminating extraneous information, which prepares the data for analysis.

Step 3: data analysis methods. Adopting a systematic approach to data analysis, this research employs various methods, including sentiment analysis, topic modeling, and time series analysis, to derive meaningful patterns from the preprocessed data (55, 56). Initially, sentiment analysis is conducted using the SnowNLP natural language processing tool, specifically focusing on the sentiment intensity regarding prepared dishes. Subsequent to sentiment analysis, neural topic modeling, utilizing BERTopic model suitable for comment texts, is applied to cluster topics for negative comments on prepared dishes. The final analytical step involves employing a time series model to examine fluctuations in sentiment and topics. In this case, it could facilitate a discussion on changing public opinions towards prepared dishes.

### 2.2 Data collection

Plenty of social media comments about the Chinese public opinion on the health effects of prepared dishes should be collected to reach the research goal. The first step involves identifying appropriate social media platforms as data sources. Considering China’s emphasis on data sovereignty, the adoption of strict data control policies such as Cybersecurity Law of the People’s Republic of China (57), the development of Internet protection protocols within physical territorial boundaries by “splinternet,” which restrict Internet access for Chinese citizens to social media platforms in other countries (58, 59), and the varied preferences in social media usage, this research draws data from six mainstream Chinese social media platforms, as detailed in Table 1. Among them, Weibo, established in 2009, is an early internet social platform allowing users to share succinct, real-time updates. According to Weibo’s financial report for the third quarter of 2023, it boasted 593 million monthly active users. Toutiao, renowned for its data mining-based recommendation engine, reports 260 million monthly active users. Xiaohongshu, a platform fostering community content, has seen its active monthly paying users exceed 200 million since 2023, with 70% being post-1990s individuals. Douyin and Kuaishou, leading China’s short video publishing sector, reports 730 million monthly and 654 million active users until 2024. Bilibili, a video community popular among the youth, averages 336 million monthly active users (60). People could share their views, attitudes, information, and ideas at any time through the above platforms, sending out text, pictures, and videos; the users could also repost these contents with the option of adding their comments.

**Table 1:** The summary of comments collection and preprocessing from social media platforms.

Social media platform	Collected comments	Preprocessed comments	Percentage (%)
Weibo	48,021	18,370	9.1
Toutiao	90,709	38,858	19.3
Xiaohongshu	32,584	12,673	6.3
Douyin	236,619	89,384	44.5
Kuaishou	115,482	21,789	10.8
Bilibili	82,785	19,919	9.9
Total	606,470	200,993	100.0

Upon selecting the social media platforms and data types, the data collection methodology warrants further consideration. The web crawler, an automated tool for web information retrieval, has been utilized to extract social media data effectively (61–63). It navigates within defined boundaries to isolate pertinent information, discarding irrelevant content (64). Starting from a specific URL, the crawler accesses linked URLs to collect and parse valuable data from each page. Utilizing the Requests3 library, the crawler navigates search pages within set parameters, receiving HTML files from the internet, which are then locally saved (65). The keyword “预制菜” (yuzhicai) was chosen to refine the search and tailor the content crawling process. The resulting dataset comprises comments with essential attributes like “created_at” (comment timestamp), “text” (comment content) and platform_name, facilitating the collection and storage of data pertinent to prepared dishes. Subsequently, this study uses Python crawler programming to capture 606,470 public social media comments about prepared dishes from 13 July 2022 to 25 March 2024, as shown in Table 1.

### 2.3 Data preprocessing

#### 2.3.1 Noise data cleaning

In the process of data collection from various social media platforms, the acquisition of some invalid and incorrect data is inevitable due to the inherent diversity and unstructured nature of these sources. This can result in the initial dataset being non-standard and unsuitable for direct analysis. Consequently, in alignment with methodologies outlined in prior research (40, 66), this study employed Python 3.10 to preprocess and cleanse a dataset comprising 606,470 original comments. This step was crucial to eliminate irrelevant content and rectify inaccuracies, ensuring the integrity of the subsequent analysis. The data cleaning process encompassed several key actions: (1) Elimination of invalid content: The dataset was purged of comments containing spam, irrelevant links, excessive punctuation, emoticons, and special characters, which do not contribute to the study’s objectives. A web cleanup technology was employed to systematically filter out such content, ensuring that only relevant data was retained for analysis. (2) Correction of typographical errors: Given the informal nature of communication on social media platforms, typographical errors are common. These errors were identified and corrected to maintain the linguistic accuracy of the dataset. This step is essential for ensuring the reliability of text-based analysis. (3) Synonym substitution: The Chinese language is characterized by a rich synonymy, which can introduce ambiguity into text-based data. To enhance the efficiency of the text mining model and reduce potential ambiguities, synonyms within the comments were standardized according to the context provided by the original data.

#### 2.3.2 Text preprocessing

Comments on social media platforms tend to be colloquial, lacking standardized structure and format. To facilitate meaningful analysis, the data must undergo extensive preprocessing, which includes: (1) Field unification: Due to varying data formats across social media platforms, it was necessary to standardize field names for essential elements like comment content, topics, and timestamps during collection. This harmonization ensures consistency across the dataset, making it more manageable and analyzable. (2) Word segmentation: For this task, we utilized Jieba, a Chinese text segmentation tool within the Python programming framework. Jieba offers a comprehensive dictionary for segmenting Chinese text into meaningful phrases. Importantly, it allows for dictionary customization, enabling the inclusion of specialized terms not present in the default dictionary. In this study, culinary-related terms were added to enhance the precision of text analysis in the context of prepared dishes. (3) Removal of stop words: The study employed a stop word list to filter out irrelevant words from the comments. Stop words, such as “this” and “that,” are commonly excluded in natural language processing to conserve storage and enhance the efficiency of information retrieval systems (67, 68).

Through above data preprocessing steps, the raw data was transformed into a unstructured corpus reflecting public attitudes towards prepared dishes, comprising 200,993 comments as detailed in Table 1. This unstructured dataset serves as a foundation for the subsequent analysis, ensuring both the relevance and the quality of the insights derived.

### 2.4 Data analysis methods

Natural language processing (NLP), a subset of machine learning, enables researchers to analyze, manipulate, and potentially generate human language. This study employs text-mining techniques such as sentiment analysis, topic modeling and time series model to automatically identify and classify patterns within large datasets, generating insights from unstructured text corpora.

#### 2.4.1 Sentiment analysis

Sentiment analysis, also known as opinion research, investigates linguistic features in terms of sentiment. It is a process that uses text analysis and computational linguistics to systematically identify, extract, quantify, summarize, and analyze subjective texts with sentimental overtones, helping researchers to elucidate the relationship between the formation of public opinions (29, 41, 45) and events (55, 69). At present, the methods of sentiment analysis mainly include dictionary-based and rule-based methods, as well as methods based on statistical machine learning (42). Dictionary-based sentiment analysis primarily involves training a sentiment dictionary. This paper utilizes the National Taiwan University Semantic Dictionary (NTUSD) and HowNet Dictionary, both widely used for sentiment analysis in Chinese, to develop positive and negative sentiment dictionaries and investigate public sentiments towards prepared dishes.

**Figure 2:**
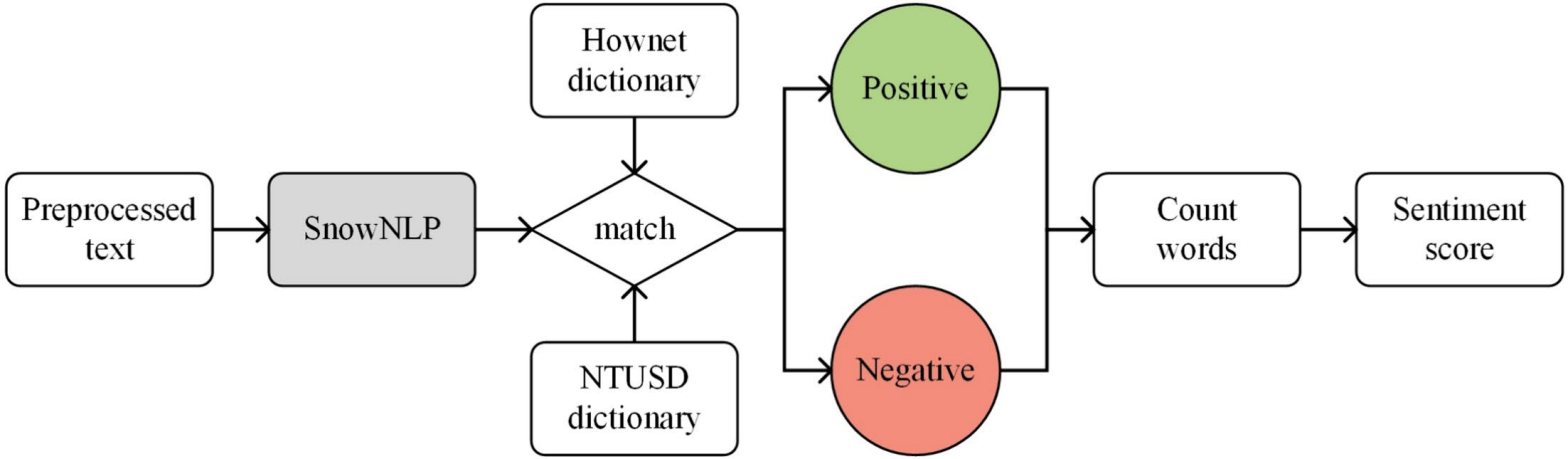
Flowchart of SnowNLP.

The sentiment value calculation and sentiment orientation assessment employ the SnowNLP library, a Python-based natural language processing component, has been widely applied in sentiment analysis research of social media texts (40, 42). The workflow of SnowNLP, as shown in Figure 2, first passes the Chinese text corpus to the module. Subsequently, SnowNLP will traverse the words in the text and look for any matches in the sentiment dictionary. Once matched, the emotional polarity (positive or negative) of the words will be determined and the number of positive and negative emotional words in the text will be calculated. In order to analyze emotions more accurately, SnowNLP also considers the relationships between words and sentence structures, such as the influence of negative words. Calculate sentiment scores based on the number, polarity, and context of emotional vocabulary SnowNLP to represent the intensity of emotions.

#### 2.4.2 Topic modeling

The endeavor of topic modeling involves leveraging statistical techniques to unearth the latent semantic frameworks within extensive text corpora (70). Grasping the central topics present in an array of texts, encompassing news articles, social media contributions, and various commentaries, furnishes valuable perspectives and strategic intelligence crucial for managerial decision-making processes across diverse sectors (29, 47, 50). This analytical approach not only demystifies the content’s inherent topics but also facilitates a nuanced understanding, enabling organizations to navigate through the informational deluge with informed precision.

**Figure 3:**
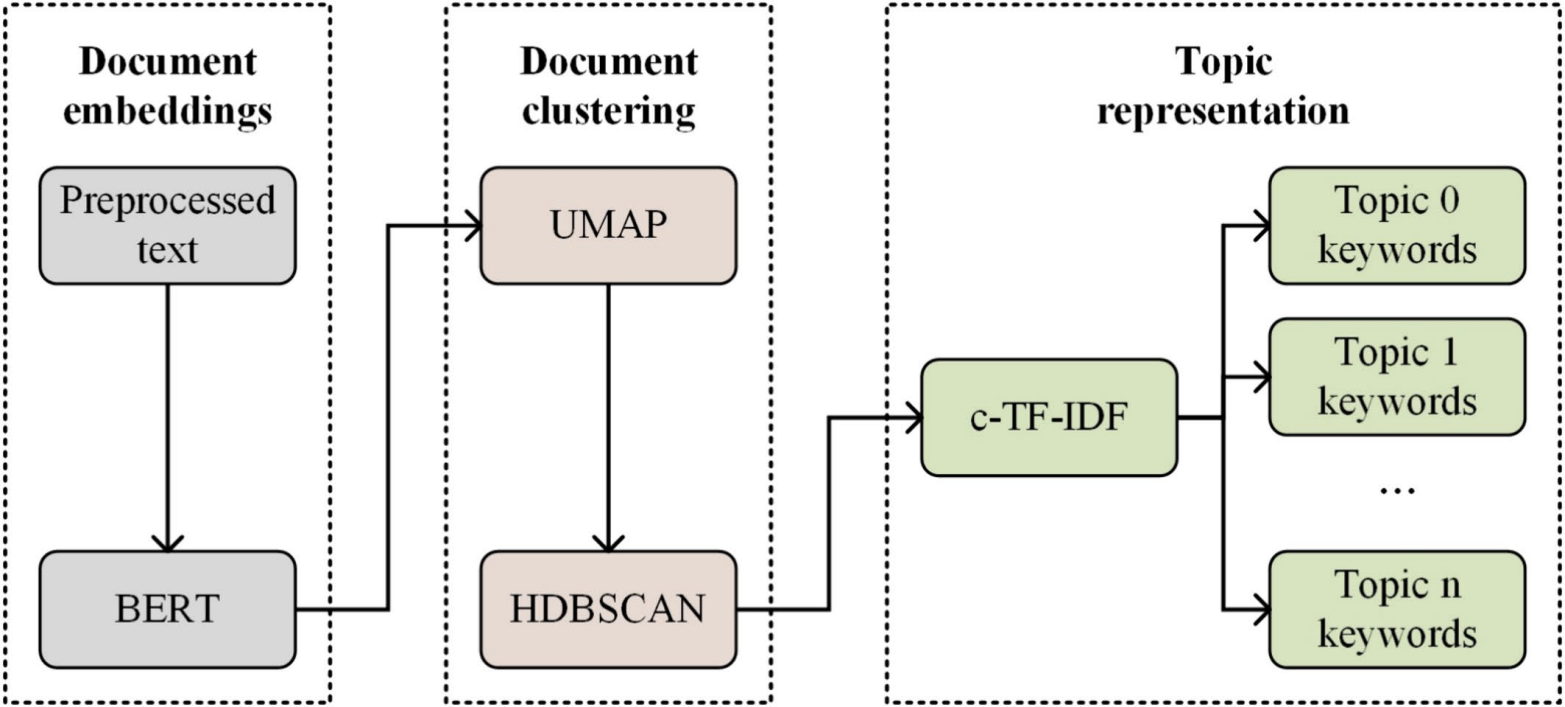
Workflow of BERTopic.

The classical approaches to topic modeling predominantly encompass Latent Dirichlet Allocation (LDA) and Probabilistic Latent Semantic Analysis (PLSA), which have been recognized as the most prevalent methodologies in the field (71, 72). Despite their widespread adoption, these models exhibit several limitations, including susceptibility to stop words, disregard for word order, the prerequisite of pre-calculating the optimal number of topics, and a lack of suitability for analyzing short texts such as social media comments. In response to these challenges (73), introduced BERTopic, a novel approach that leverages BERT embeddings alongside c-TF-IDF to generate dense clusters, facilitating the interpretation of topics and ensuring the preservation of significant words within topic descriptions. This methodology has demonstrated considerable efficacy in topic modeling across diverse domains (74–77). Consequently, as depicted in Figure 3, the present research endeavors to apply the BERTopic model to the analysis of social media comments pertaining to prepared dishes, aiming to mine the underlying topics. BERTopic generates coherent topics through follow steps:

1. Document embeddings. BERTopic uses the Sentence-BERT (SBERT) framework. Therefore, in the process of mining social media comments related to prepared dishes, BERT embedding word vectors are first used, which preserve context sensitivity and relationships, and then represent the comment text as points or vectors in a continuous vector space.
2. Document clustering. First, the Uniform Manifold Approximation and Projection (UMAP) is to reduce the dimensionality of embeddings, has been shown to better preserve both local and global data features, which has three calculation processes.

- Constructing the K-Nearest Neighbor (KNN) graph. For each point m in a high-dimensional dataset, find its nearest K nearest neighbors. This can be achieved by calculating the distance between point *m* and all other points in the dataset, and then selecting the nearest K points. For point m and one of its nearest neighbors n , the distance d(m,n) can be calculated using Euclidean distance, illustrated in Equation 1.

(1) $$d\left(m,n\right)=\sqrt{\overset{D}{\underset{i=1}{\sum }}{\left(x_{m_{i}}-x_{n_{i}}\right)}^{2}}$$

Where D epresents the dimensionality of the data, $x_{m_{i}}$ and $x_{n_{i}}$ are the coordinate values of point *m* and *n* in the i -th dimension, respectively.

- Calculating high-dimensional joint probabilities. In order to establish similarity relationships between high-dimensional samples, UMAP adopts a joint probability distribution. The calculation process is shown in Equation 2.

(2) $$P\left(n/m\right)=\frac{\exp\left(-d\left(m,n\right)/\sigma_{m}\right)}{\sum_{k\neq m}\exp\left(-d\left(m,n\right)/\sigma_{m}\right)}\;$$

The joint probability P(n/m) is computed based on the distance between point m and its neighbors, with $\sigma_{m}$ serving as a local scale parameter that adjusts the sensitivity of distances within the neighborhood of point m .

- Adjusting for probability consistency. After the joint probability calculation of all comment data points is completed, in order to ensure the consistency of probabilities between any two data points, an expression is introduced in Equation (3).

(3) $$B=A+A^{T}-A⊙A^{T}$$

Among them, A represents the weighted adjacency matrix composed of all E(m,n) , ⊙ represents the Hadamard product of the matrix, and B is the modified weighted adjacency matrix, used to ensure consistency and symmetry between data points.

Subsequently, the reduced embeddings are clustering used the Hierarchical Density-based Spatial Clustering of Applications with Noise (HDBSCAN), an advanced version of DBSCAN that using a soft-clustering approach allowing noise to be modeled as outliers. This prevents unrelated documents to be assigned to any cluster and is expected to improve topic representations, which has two calculation processes.

- Modeling distance probabilities in low-dimensional space. In the UMAP algorithm, the distance probability modeling of low dimensional space is achieved through the curve family $\frac{1}{1+s\cdot c\left(2t\right)}$ , where s and t are hyperparameters used to adjust the sensitivity of the model and maintain the topological structure. The probability function constructed in low dimensional space is specifically expressed in Equation (4).

(4) $$F\left(m,n\right)={\left(1+s{\left(y_{m}-y_{n}\right)}^{2t}\right)}^{-1}$$

Among them, $y_{m}-y_{n}$ represents the distance between any two data points $y_{m}$ and $y_{n}$ in a low dimensional space, reflecting the relative positional relationship between comment data points after dimensionality reduction.

- Cross-entropy loss function. In order to ensure that the reduced dataset can maintain the structural characteristics of the original dataset as much as possible, it is necessary to optimize by minimizing the difference in similarity distribution between data points in high-dimensional space and low dimensional space. This difference can be quantified through the cross entropy loss function, which is used to measure the similarity between two probability distributions. Its specific expression is shown in Equation (5).

(5) $$L=-\underset{m,n}{\sum }\left[\begin{array}{l} E\left(m,n\right)\log\left(F\left(m,n\right)\right)+\\ \left(1-E\left(m,n\right)\right)\log\left(1-F\left(m,n\right)\right) \end{array}\right]$$

In this expression, E(m,n) represents the joint probability between data points m and n in high-dimensional space, while F(m,n) is the simulation probability between corresponding points in low dimensional space. By optimizing this loss function, points in low dimensional space can be adjusted to better reflect the data structure in high-dimensional space, especially in maintaining the relative distance and distribution pattern between data points.

1. Topic Representation. The c-TF-IDF was used to evaluate the degree of importance of words within a clustered cluster, generates topic representation. The c-TF-IDF of a single word in topic *t* was calculated. The frequency of each word w for each topic t ( $w_{t}$ ) was divided by the total number of words n ( $n_{t}$ ) in the documents of topic t , which is the extension of TF-IDF. Subsequently, the average number of words *m* in each topic *t* was divided by the total frequency of word w across all i topics. The calculation of c-TF-IDF is shown in Equation (6).

(6) $$c-TF-IDF_{t}=\frac{w_{t}}{n_{t}}\times \log\left(1+\frac{m}{\sum_{j=1}^{i}w_{j}}\right)$$

#### 2.4.3 Time series model

Time series data can reflect the dynamic characteristics of the described phenomenon over time. Peoples’ sentiments orientation and discussed topics towards the health effect of prepared dishes can be reflected in publics’ opinion, including positive or negative sentiments and the main topic focus of attention, and their changing trends for prepared dishes. Then, continuous data collection across time allows time-series analysis, which is significant in exploring a corpus for expressing public opinions of longitudinal data. In order to better understand publics’ opinion to various topics and sentiments changes of prepared dishes, this paper employs the time-series concept to divide the research period into time intervals using the captured social media comments, refers to the previous research (40, 78) and adopts the Mann-Kendall method to analyze the attitude changes of prepared dishes. The Mann-Kendall trend test is a non-parametric test method proposed by Mann and Kendall (79, 80), also known as the distribution-free test. It is suitable for analyzing time series with continuous increasing or decreasing trends (monotonic trends) whose advantage is sample data does not need to satisfy the assumption of normal distribution, nor is it disturbed by individual outliers. Therefore, it is widely used in academic research related to trend testing.

As follow, set a time series X as the statistic H in the Mann-Kendall test. The calculation of H is shown in Equations (7) and (8).


(7) $$H=\overset{k-1}{\underset{f=1}{\sum }}\overset{k}{\underset{p=f+1}{\sum }}\mathrm{sgn}\left(x_{f}-x_{p}\right)\;$$

(8) $$\mathrm{sgn}\left(x_{f}-x_{p}\right)=\{\begin{array}{cc} 1 & if\;x_{f}-x_{p}>0\\ 0 & if\;x_{f}-x_{p}=0\\ -1 & if\;x_{f}-x_{p}<0 \end{array}$$

Among them, k represents the total number of time series data points, $x_{f}$ represents the data point of former time, and $x_{p}$ represents the data point of present time.

The following formula can be obtained by standardizing the statistic H , as shown in Equation (9).


(9) $$C=\{\begin{array}{cc} \frac{H-1}{\sqrt{\mathrm{var}\left(H\right)}} & if\;H>0\\ 0 & if\;H=0\\ \frac{H+1}{\sqrt{\mathrm{var}\left(H\right)}} & if\;H<0 \end{array}\;$$

The statistic C obeys a standard normal distribution. If the *p*-value is less than the significance level (α=0.05) , it indicates a significant increasing or decreasing trend.

## 3 Results and discussion

### 3.1 Results

#### 3.1.1 Public sentiment analysis of prepared dishes

**Figure 4:**
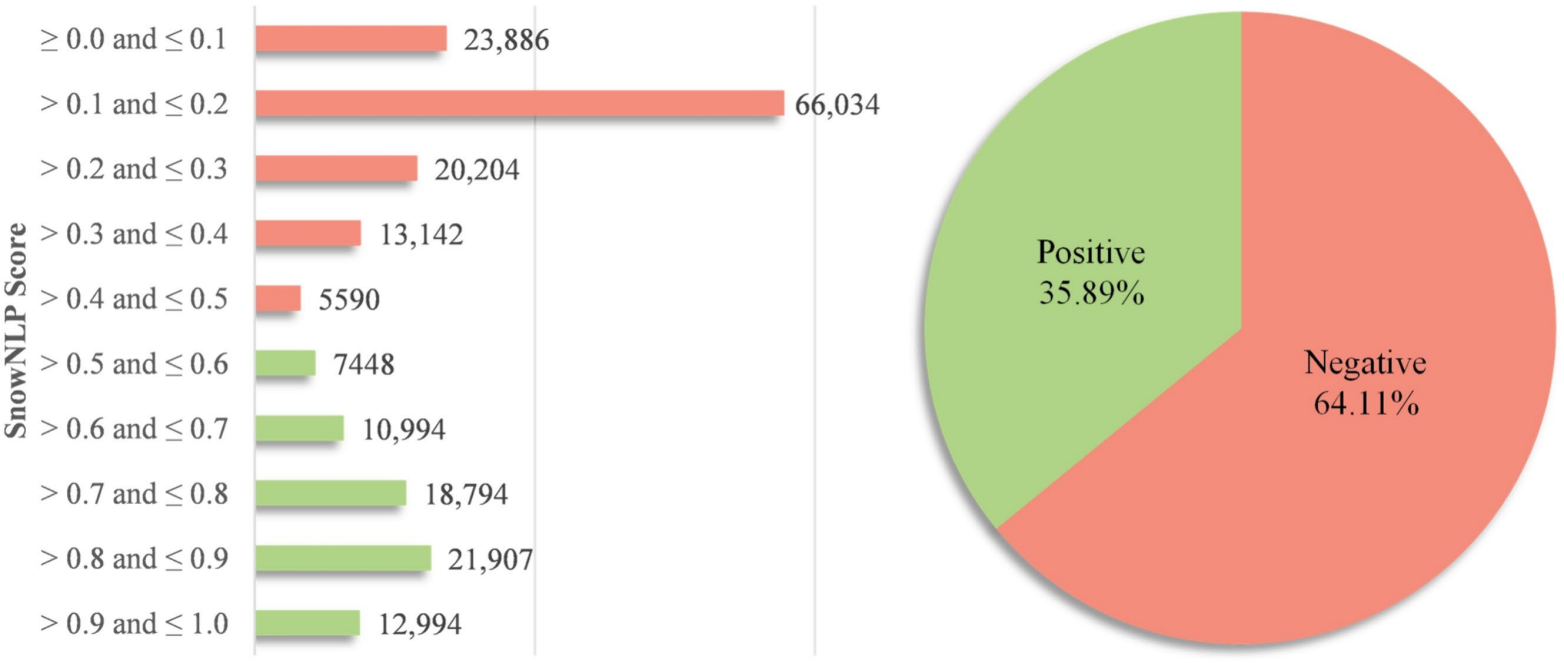
Sentiment distribution of all comments.

Following the preprocessing procedure, this study obtained a total of 200,993 eligible comments, which were then subjected to sentiment scoring using SnowNLP. SnowNLP assigns scores ranging from 0 to 1, representing the probability that a comment has a positive sentiment. In this study, comments scored within the range of [0, 0.5] were classified as negative sentiment, while those scored in the range of [0.5, 1] were classified as positive sentiment. Public sentiment orientation on prepared dishes is show in Figure 4; negative comments accounted for 64.11%, while positive comments took up 35.89%. It is evident that the Chinese public holds a majority of negative sentiments towards prepared dishes.

Then, in Figure 4, the left side shows the sentiment distribution across various score segments, revealing a significant disparity in how sentiments are distributed. This detailed analysis of sentiment scores highlights the varying degrees of emotional expression among the comments. Concerning the comments with negative sentiments, a substantial majority of the comments fall within the negative range of [0.0, 0.2], suggesting a strong prevalence of negative emotional expressions regarding prepared dishes. Conversely, the positive sentiments, primarily found within the range of (0.6, 0.9]. This can be attributed to a substantial portion of individuals acknowledging the benefits of prepared dishes, such as convenience, speed, and ease of storage (16, 19). Additionally, given China’s large population, the prepared dish industry is poised to become a substantial sector with numerous stakeholders (17, 18, 20).

It is generally known that people exhibit negative emotions when voicing concerns about their health (22). Consequently, it becomes essential to delve deeper into these expressions, particularly focusing on topics related to public health present within the negative comments. Therefore, in this step, a total of 128,856 negative sentiment comments related to prepared dishes have been identified. The next step will involve analyzing topics related to public health within these negative comments.

#### 3.1.2 Topic analysis of negative comments

In the second step, the BERTopic model was employed to conduct a cluster analysis of 128,856 negative sentiment comments. This study identified and analyzed the 50 most frequently occurring topics, labeled Topic0 to Topic49. Topics beyond Topic49, starting from Topic50, exhibited a drop in the proportion of negative affective comments to less than 2%. Given this minimal contribution to the study’s objectives, these topics were deemed peripheral and consequently excluded from further analysis.

Subsequently, to focus on the effects of the Chinese public on the health implications of prepared dishes, it is necessary to select health-related topics from the first 50 topics for further analysis. Due to the inherent variability and richness of language used in social media comments, where multiple expressions may convey similar meanings, this study chose to respect the diversity and complexity of such language by not standardizing or altering it. This approach acknowledges the natural arbitrariness and richness of user-generated content, allowing for a more authentic analysis of sentiment and expression. The selection of topics for analysis adheres to several guiding principles: (1) The topics must contain keywords explicitly related to the production and consumption of prepared dishes, or directly pertain to health, disease, and potential harm, ensuring relevance to the core subjects of investigation. (2) Due to the complex nature of the production and consumption chains of prepared dishes, topics that include keywords associated with these processes are also incorporated. Special emphasis is placed on scenarios linked to educational settings and adolescent health, recognizing the heightened sensitivity and potential impacts in these areas. (3) Topics concerning market supervision, regulatory measures, and the enforcement of penalties for production and marketing practices detrimental to health are also selected. This inclusion reflects the importance of governance and accountability in safeguarding public health within the prepared dish industry.

**Figure 5:**
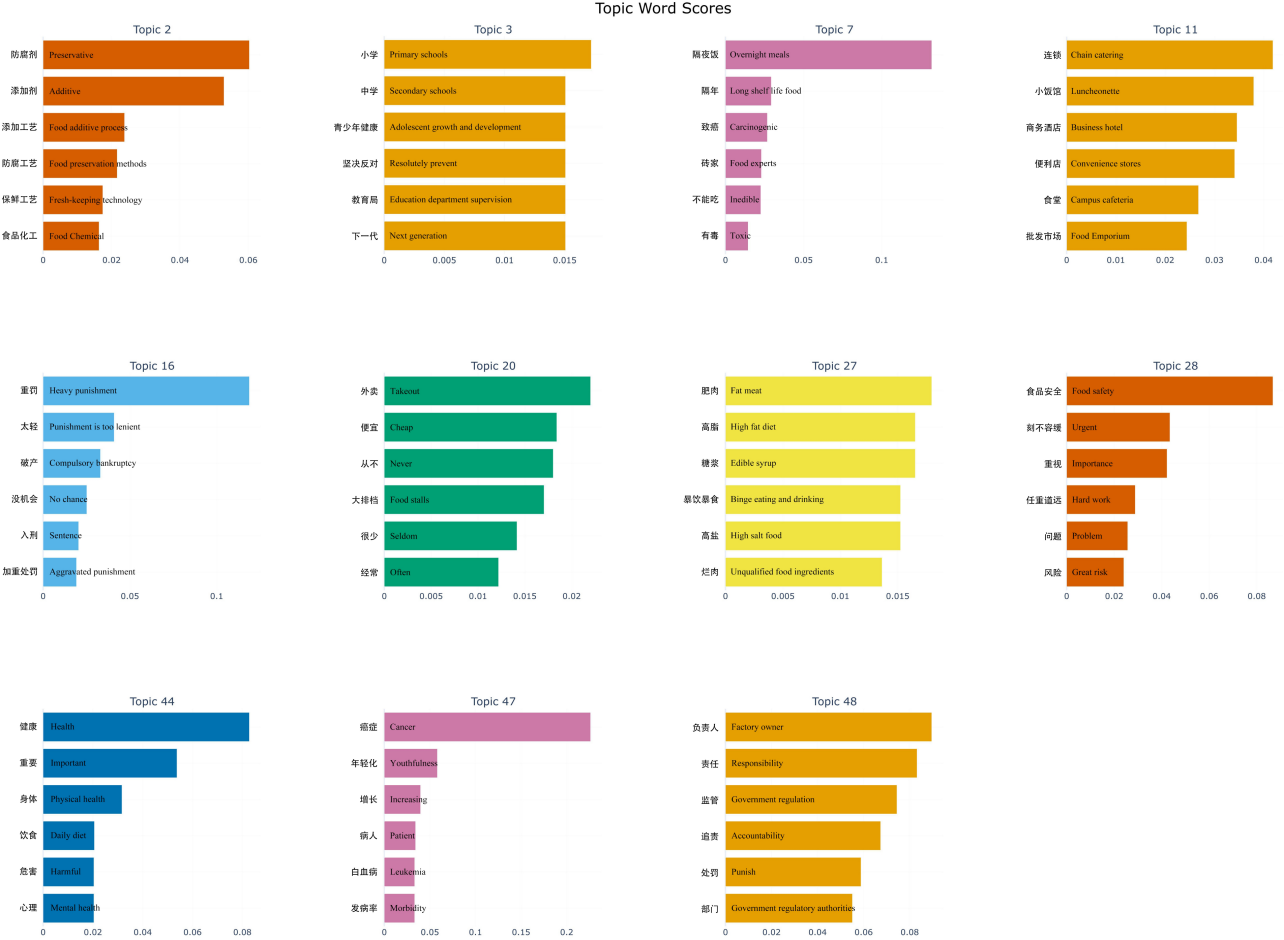
BERTopic clustering towards health effects of prepared dishes.

**Figure 6:**
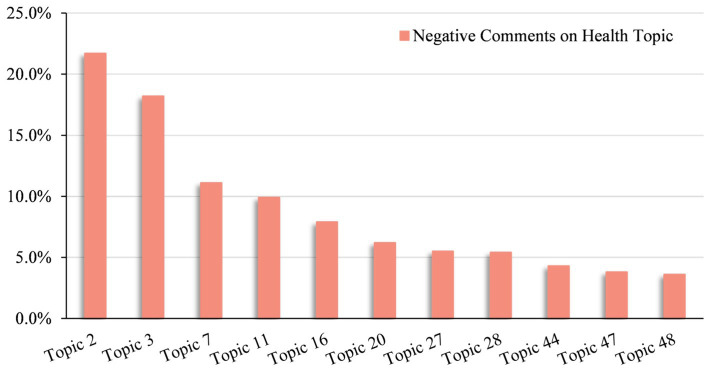
Proportion of negative comments on health topic.

Based on the above principles, 11 topics were found to be closely related to public health concerns, as depicted in Figure 5. Meanwhile, the proportion of these 11 health-related topics in all negative affective comments is shown in Figure 6.

For the 11 selected topics, an analysis of the corresponding keywords under each topic provides insight into the public’s concerns about the health impacts of prepared dishes. Figure 5 illustrates several key findings:

Firstly, Topic 2, the third most discussed topic, includes keywords such as “Preservative, Additive, Food chemical,” which reflect people’s doubts about the health effects of using preservatives and various additives in the production of prepared dishes. Then, Topic 7 features words like “Overnight meals, Inedible, Toxic,” emphasizing concerns about the safety and health implications of the long-term storage process typical of prepared dishes. Topic 27 includes terms such as “Fat meat, High salt food, Edible syrup, Unqualified food ingredients,” indicating public concerns over the use of ingredients that are high in sugar and fat, and even potentially unsafe animal tissues in pre-packaged food products. These three topics reflect legitimate people concerns arising from the technical aspects of prepared dishes and the potential health issues associated with their production processes.

Furthermore, by analyzing keywords such as “Adolescent growth and development, Resolutely prevent, Physical health, Harmful, Mental health, Cancer, Leukemia” in Topics 3, 44, and 47, it becomes apparent that people are discussing the detailed impact of prepared dishes on health, linking an increasing incidence rate of cancers and other diseases in young people to these products.

Additionally, Topics 3, 11, and 20 address the environments where people encounter prepared dishes, with keywords like “Primary schools, Secondary schools, Chain catering, Luncheonette, Takeout, and Food stalls.” These discussions reveal that prepared dishes are commonly found in small restaurants, chain convenience stores, and food stalls, with takeout being the most prevalent distribution method. There is particular concern over the extensive use of prepared dishes within the canteens of public primary and secondary schools, which has elicited strong negative reactions.

Then, according to the third principle of topic selection, keywords such as “Food safety, Urgent, Importance, Heavy punishment, Factory owner, Government regulation, and Accountability” found in Topics 28, 16, and 48 underscore significant concerns about the impact of prepared dishes on health. These topics identify the potential harms of prepared dishes as stemming from a perceived lack of corporate social responsibility and the profit-driven moral degradation of business owners. Consequently, there is a public demand for increased media oversight to prevent unethical business practices and for government regulatory bodies to intensify the severity of penalties for such malpractices. There is also expressed dissatisfaction with the current laws and regulations that govern these enterprises.

**Figure 7:**
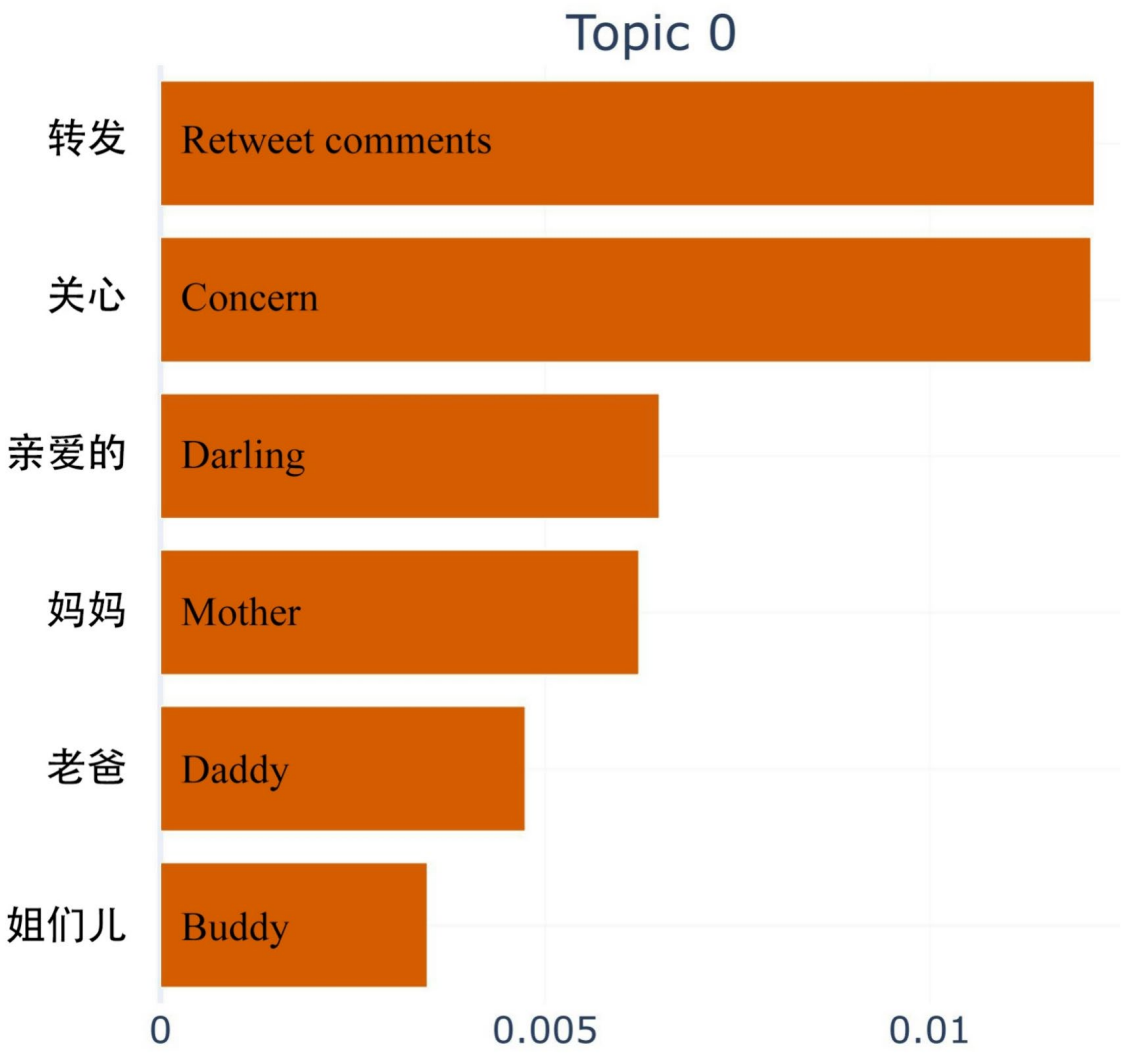
Ranking first topic.

Finally, although Topic 0 was not selected as a health-related topic, it ranks first and needs to be discussed separately in this study. In Figure 7, Topic 0 corresponds to the importance level of the corresponding keyword, and “Retweet comments” ranks first, indicating that a significant people belief that prepared dishes pose health risks. Retweeting these concerns to friends and relatives is seen as a protective measure in situations where individuals feel alarmed and powerless to effect change directly.

An equally noteworthy experimental result is the heatmap of negative comments on the health effects of prepared dishes, which provides relevant information on public focus areas regarding the health effects of prepared dishes. This tool helps establish an intuitive understanding of the relationship between these focus topics.

**Figure 8:**
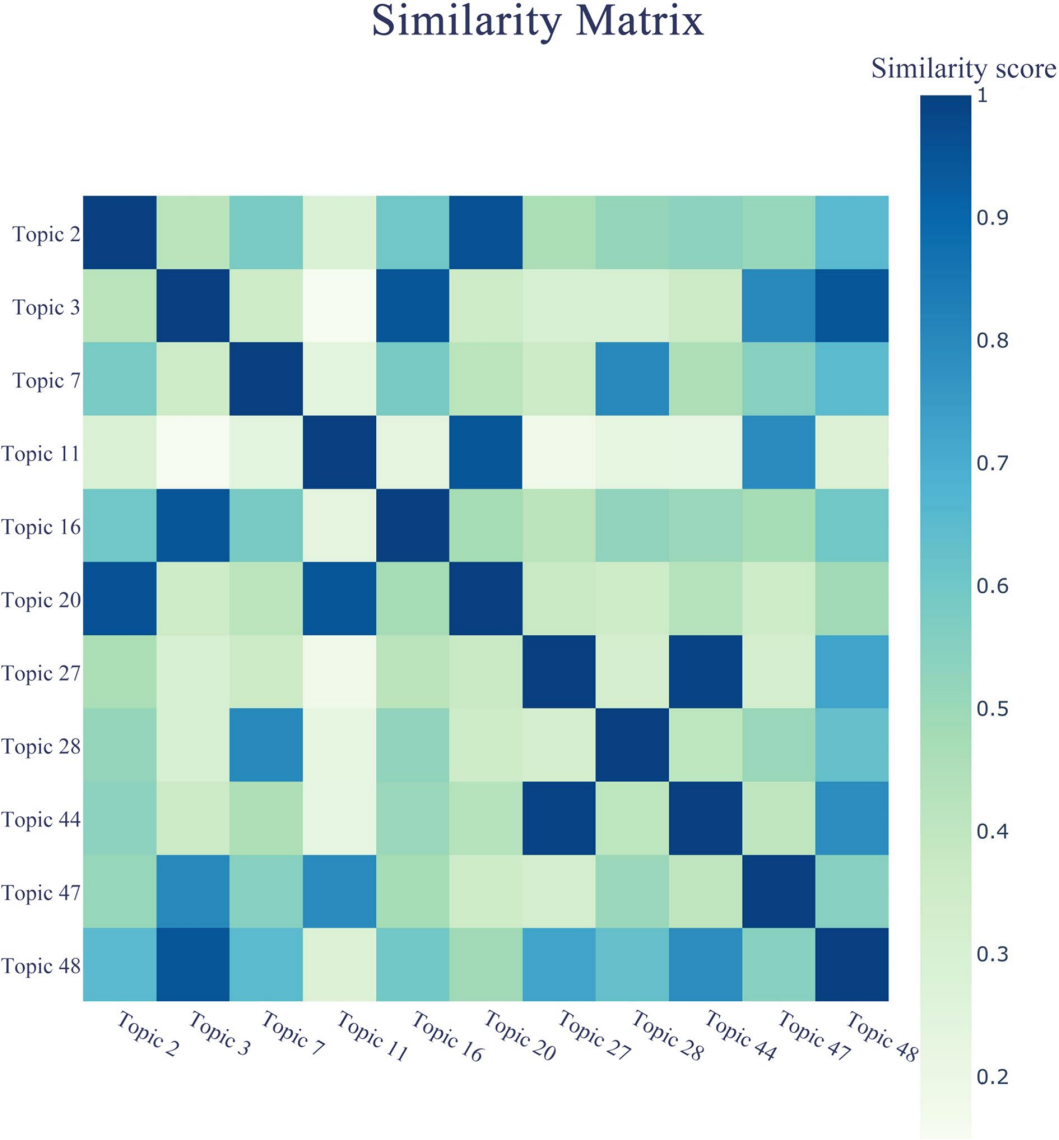
Heatmap of health effects topics.

In Figure 8, the depth of colors indicates the degree of correlation or similarity between topics. Topic 27 includes words such as “Fat meat, High salt food, Edible syrup, Unqualified food ingredients,” while Topic 44 contains phrases like “Health, Important, Physical health, Daily diet.” With a correlation score of 0.9, this suggests that the public generally believes prepared dishes, which are high in fats, sugars, oils, and salts and contain low-quality ingredients, pose a serious threat to physical and mental health. Similarly, Topics 2 and 20 include terms such as “Preservative, Additive, Food chemical, Takeout, Cheap, Never, and Food stalls,” with the same high correlation score of 0.9. This indicates that the production and processing technologies of prepared dishes are closely linked to the environments in which they are produced, significantly impacting public health perceptions and influencing negative public comments.

Furthermore, the correlation score between the phrases “Primary schools, Secondary schools, Adolescent growth and development, Resolutely prevent” in Topic 3, “Factory owner, Responsibility, Accountability, Punish” in Topic 48, and “Heavy punishment, Compulsory bankruptcy, and Sentence” in Topic 16 reached 0.8. This demonstrates strong public opposition to the introduction of prepared dishes on school campuses and a pressing call for government departments to strictly regulate companies that fail to adhere to regulations. The aim is to prevent the introduction of prepared dishes into schools through administrative measures such as accountability and punishment, minimizing their impact on youth health.

Lastly, Topics 11, 20, and 47, with a correlation score of 0.7 and including keywords such as “Chain catering, Luncheonette, Business hotel, Takeout, Cheap, Never, Cancer, Youthfulness, Increasing,” reveal that in locations such as chain restaurants, small restaurants, and business hotels—where dining out is common—prepared dishes frequently appear on dining tables. The ubiquity of prepared dishes in physical restaurants has penetrated daily meals, raising fears that long-term consumption may cause serious health issues, including cancer. Additionally, the correlation between the terms “Overnight meals, Carcinogenic, Inedible, Toxic” in Topic 7 and “Food safety, Urgent, Importance, Great risk” in Topic 28 is 0.7. This reflects public concern that the long-term storage of prepared dishes may produce carcinogenic and toxic substances that jeopardize health, underscoring the urgent need to address prepared dishes within the scope of food safety management.

In summary, the correlation analysis of the health impact of prepared dishes on related topics comprehensively interprets the public’s real and objective reactions to prepared dishes food, including their cognition, concerns, and appeals, from the lexical level to the thematic level.

#### 3.1.3 Sentiment and topic changes analysis of based-time series

**Figure 9:**
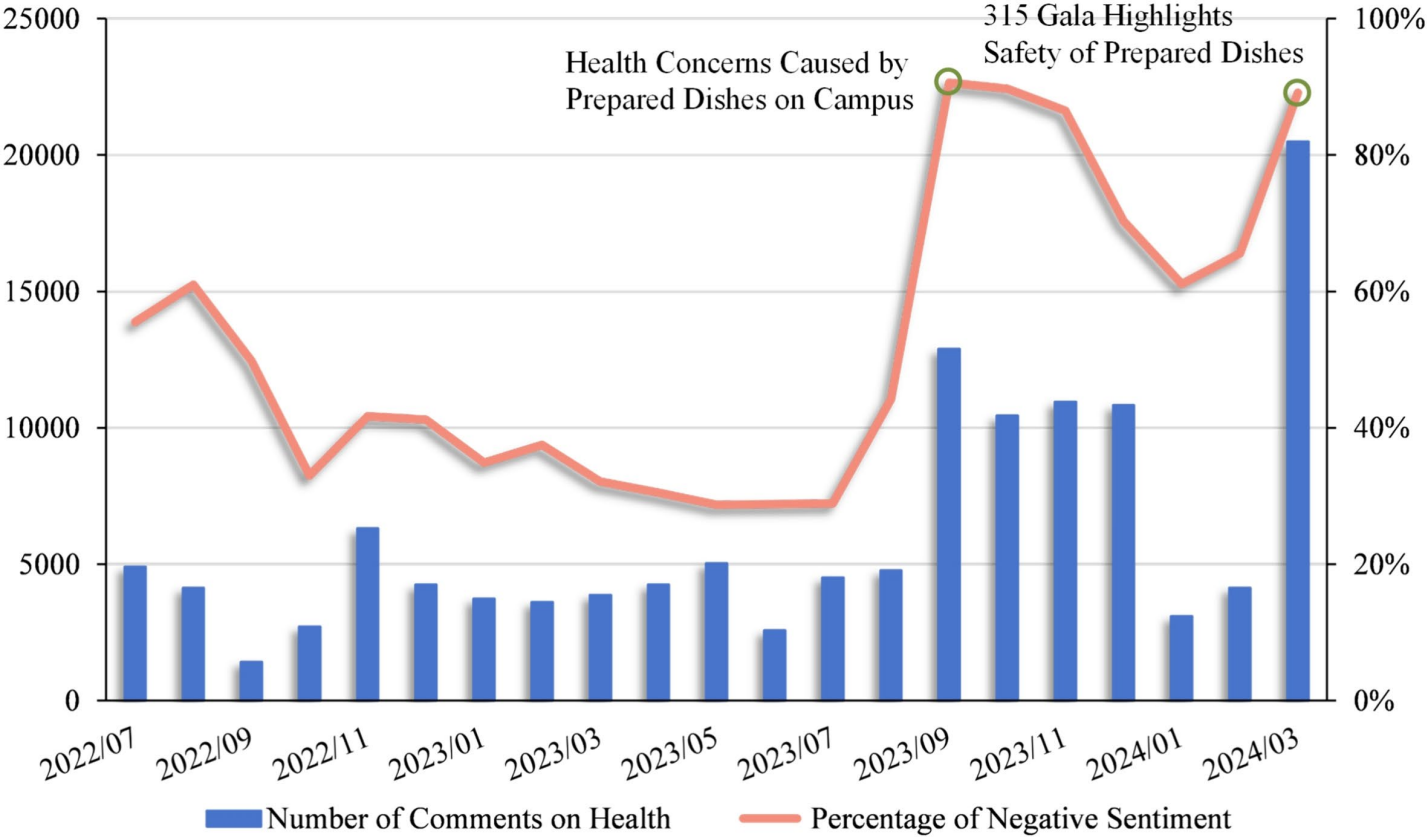
Number of comments on health and percentage of negative sentiment per month.

Figure 9 illustrates the changes in the number of prepared dishes-related comments and the percentage of negative sentiment per month from July 2022 to early March 2024. From 2022 to July 2023, the number of social media comments discussing the health effects was relatively small, averaging no more than 5,000 comments per month. During this period, the proportion of negative sentiments averaged no more than 50%, indicating that the public maintained a relatively neutral stance towards prepared dishes when discussing them on social media platforms, with limited discussion about their impact on health. This trend may be attributed to the “No. 1 Central Document issued in February 2023,” which focused on cultivating and developing the prepared dishes industry, possibly leading to fewer health-related comments and more neutral emotions.

Since then, the social media comments number on health effects of prepared dishes has had noticeable fluctuation, but an overall upward trend can be observed. Notably, comments engagement peaked in September 2023, coinciding with the start of the school year. At this time, some primary and secondary schools in parts of China began introducing prepared dishes, sparking heated discussions and strong negative reactions due to concerns over the health implications of such meals. The related event “Health concerns caused by prepared dishes on campus.”

Subsequently, in January 2024, as depicted in Figure 9, comments related to health decreased, and the intensity of negative emotions softened. This change demonstrates the extensive social discussion and the previously strong negative sentiments concerning the use of prepared dishes in schools. Chinese education authorities have adopted a cautious approach regarding the use of prepared dishes in schools and implemented corresponding regulatory measures, as indicated by the incident “Prohibition of prepared dishes in primary and secondary schools across various provinces and cities.” However, these measures had little effect on reversing the public’s negative perceptions of prepared dishes, with negative sentiment still exceeding 60% at that time.

Finally, it is evident from Figure 9 that the number of comments and the proportion of negative emotions related to the health of prepared dishes reached their peak in March 2024. The corresponding event is “March 15, 2024, China’s annual consumer rights protection day.” Some official media reports on certain manufacturers illegally producing processes and using raw materials have once again raised public concerns and strong negative emotions about the health impact of prepared dishes.

Overall, the fluctuations in the number of comments and sentiments across different events reveal the sensitivity of the Chinese public to issues related to prepared dishes. Positive publicity often leads to suspicion and negative reactions, while cautious policies and government actions often receive public support.

### 3.2 Discussion

In contrast to the traditional self-report method, this study adheres to a data-driven research paradigm (51, 53, 81), subjecting the data to strict cleaning and preprocessing. The primary purpose was to identify objective public expressions within social media comments, thereby establishing a semantic analysis framework encompassing “Sentiment Analysis—Topic Mining—Time Series Analysis.”

Initially, sentiment analysis of 200,993 social media comments disclosed that approximately 64% exhibited negative emotion, reflecting public opposition to prepared dishes. This finding, however, differs from prior research. For example, a study focused on consumers’ cognition and purchase intentions indicated a positive attitude towards prepared dishes in supermarket interview surveys involving 1,209 participants (18). Additionally, a questionnaire survey of 1,767 respondents demonstrated that over 90% of adults accepted prepared dishes, with outdoor camping scenarios being the most favored, accounting for 52.8% (82). We believe that the difference between our research results and previous ones comes from the research based on social media data, where the expression of public opinions and questionnaire surveys are in entirely different environments. Due to the close relationship between social media and contemporary life, we believe that people can freely express their opinions on a particular topic rather than being limited by the scope of survey questionnaires. Therefore, previous studies in many fields have shown that social media comments have better objectivity, especially when the data volume and coverage are sufficient. Although expressing opinions through social media data often lacks strict comparison and reasoning processes compared to questionnaire data, it is easier to express them intuitively. More importantly, for significant news events, policies, and other external factors, social media data has higher real-time sensitivity, spreads faster, and retweets more frequently (83). Also, scholars have suggested that social media data analysis is a valuable supplement to the in-depth, context-rich insights provided by survey data analysis, with its extensive breadth and capacity to capture real-time, large-scale trends (45, 84). Previous analyses of questionnaire and interview data have indicated that the Chinese public concerns regarding the food safety and perceived health risks associated with prepared dishes (17–20). In the present study, previous research results guided the analysis based on social media comments. Consequently, the design of the semantic analysis framework focused first on identifying negative comments and then on exploring health-related topics within those negative comments, as people often express negative emotions when discussing health concerns.

Secondly, topic mining of 128,856 negative comments identified 11 topics related to the health effects of prepared dishes. Compared with a previous empirical study on the perceived health risks of prepared dishes (19), which has five questions in the designed questionnaire, our results are more fine-grained. Our study contributes to this line of research by further measuring the public’s perception of the health effects of prepared dishes using objective data derived from social media comments. Moreover, based on the 11 health-related topics identified, we ranked them by the proportion of negative comments. The top-ranked topic, Topic 2, involved keywords such as “Preservative, Additive, Food chemical,” which confirmed previous survey results showing that the public was most concerned about illegal additives in prepared dishes, accounting for 68.55% (22). Further, after interpreting the meaning of each topic and discussing the correlation between the topics through the heatmap output of the BERTopic model, we validated these findings by comparing them with previous studies on prepared dishes from different perspectives. For instance, Topic 3 highlighted concerns about prepared dishes in primary and secondary school canteens affecting students’ health. This topic ranked second in negative comments, supporting previous survey results indicating that 54.68 and 42.6% of the public do not accept prepared dishes in primary and secondary school canteens (22, 82). Similar to present studies, topics 16, 28, and 48 present the public’s spontaneous demands for punishment, supervision, and accountability for the health effects of prepared dishes, which coincide with the proposed measures in the previous research results (17, 18, 20, 85, 86). The topic mining results of this study not only prove that the social media comment data can reflect the needs and opinions in the “real world” but also present issues that have not been paid attention to in previous studies. For example, Topic 20 reveals public concern about the health impact of take-out meals and a rejection of the use of prepared dishes in such meals, an area not covered in earlier surveys (82). In particular, topic 0 is also a manifestation of the unique properties of social media data, and the keyword “Retweet comment” ranks first in the topic, which proves that negative comments are spread quickly and forwarded more on the Internet (45, 83). This explains why most health-related content in our study about prepared dishes expressed by the public on social media platforms contains negative emotions.

Last but not least, it is important to acknowledge that previous empirical or survey studies have typically covered a specific period (17–20, 22, 82), with public opinion potentially evolving dynamically over time. This temporal limitation can obscure understanding of how perceptions respond to new information or events. This study employs automated web crawling and advanced big data analytics to monitor real-time changes in public perceptions of prepared dishes. The results of our study reveal that significant events, such as “Health Concerns Caused by Prepared Dishes on Campus” and “March 15, 2024, China’s annual consumer rights protection day,” frequently capture social media attention. These events trigger a surge in comments, controversies, and expressions of negative emotions. Although social media comment data often follows intuition and lacks in-depth thinking and sufficient rational judgment, these features may introduce bias in research (83). However, they can highlight the sensitivity of social media data to event-driven fluctuations, which is consistent with the researchers’ observations. Therefore, social media data has unique advantages over traditional survey methods in fields that require high real-time performance. For instance, this approach has been successfully utilized in studying public perceptions of various food products, including alternative meats (45), organic foods (29), and coffee (44). The immediacy and breadth of data from social media allow for a more instantaneous and comprehensive observation of public opinion compared to conventional questionnaire surveys and interviews. This ability to capture real-time shifts in public sentiment is invaluable for businesses and governmental bodies. It enables a swift and nuanced understanding of emerging trends and public concerns regarding the health effects of prepared dishes. The key is that stakeholders can make wiser decisions and take timely intervention measures to address public concerns, ensuring their strategies remain relevant and effective in a rapidly changing social environment. This is very important for the Chinese government and enterprises eager to promote the development of prepared dishes.

## 4 Conclusions and implications

### 4.1 Conclusions

In response to the growing awareness of health hazards and related issues associated with prepared dishes, this study aimed to explore public perceptions regarding the health effects of these products. People are now more willing to express their opinions on social media platforms. Thus, this study used this kind of data to explore the concerns of the Chinese public about the health of the rapidly developing prepared dishes industry with a fast response time. Our study utilized public comments on social media, employing a semantic analysis framework based on NLP technology to identify and analyze the most discussed health effects of prepared dishes within negative comments and tracking their evolution over time. The findings demonstrate that individuals are more willing to express their opinions on social media platforms, free from external biases. The results obtained through semantic recognition of comment data from these platforms provide relatively objective and verifiable insights.

Academically, this research is the first early-stage study to use social media comments and NLP methods to examine public perceptions of the health effects of prepared dishes. It enriches existing literature by providing a broader and more nuanced understanding of public opinion towards prepared dishes. Unlike traditional methods, this study explores real-time observations, offering a more dynamic view of public opinion than phased observations from surveys and interviews. The results show that the public initially had concerns about several technical aspects of the prepared dishes industry, including production processes, food ingredients, additives, and packaging materials, which were perceived as potential health hazards. There was also notable distrust of companies involved in producing prepared dishes, with strong calls for increased media scrutiny and government regulation. Additionally, the act of retweeting comments further amplified public discourse, vividly expressing individual concerns about the potential health risks of these products. Practically, our findings can help policymakers and businesses strengthen the regulation of prepared dishes, ultimately building public confidence and ensuring the sustainable growth of this market. This information empowers stakeholders to make informed decisions and take actions that benefit public health and the industry.

In conclusion, this study addressed these limitations compared to previous research, which primarily relied on surveys and interviews and was often limited by factors such as small sample sizes and a lack of real-time responsiveness. Our research findings are mutually consistent with existing studies in major aspects, and they have better real-time performance and more extensive coverage. Moreover, some differences compared to previous research reflect new characteristics of changes in the expression of the public will. That is, the negative emotions of the public towards a particular product or category, especially those related to food safety and health, will quickly spread to a larger audience through social media. The characteristics of these changes suggest that government management departments, enterprises, and researchers should attach importance to such changes.

### 4.2 Implications

For the government, the role of regulatory bodies is crucial in maintaining the integrity and safety of the food industry, particularly concerning prepared dishes. With China’s rapid urbanization and changing lifestyle dynamics, governmental agencies must prioritize enacting stringent food safety regulations to protect public health, enforcing existing laws and adapting regulatory frameworks to address the new challenges the prepared dishes industry poses. Proactive government oversight is necessary to ensure that enterprises adhere to high product quality and safety standards. Moreover, government initiatives to stimulate industrial prosperity during economic downturns should integrate robust mechanisms for capturing and responding to public sentiment, ensuring that growth does not compromise food safety standards.

For enterprises and manufacturers, due to public concerns about the health effects of prepared dishes, it is imperative to prioritize aligning their operations with both public expectations and regulatory standards. This alignment is crucial in navigating the complexities of public health concerns and consumer skepticism. Manufacturers, in particular, play a pivotal role in innovating safer and more nutritious food preparation technologies that can mitigate health risks associated with prepared dishes. These entities must invest in the continuous improvement of food quality, diversification of flavors, and enhancement of nutritional values to meet the evolving demands of consumers. Additionally, fostering transparency about ingredient sourcing, production processes, and safety measures is essential to build trust and facilitate informed consumer choices.

For the general public, the advent of “we-media” has significantly transformed their role from passive consumers to active participants in the discourse surrounding food safety and industry practices. The widespread dissemination of information empowers the public to express concerns and demand transparency and accountability from food producers. The study underscores the necessity for public engagement in dialogue and decision-making processes related to food policies. By voicing their concerns, the public can influence industry practices and governmental policies, contributing to the overall improvement of food safety standards.

For the prepared dishes industry to thrive sustainably, a multi-stakeholder approach is essential. Governments must continually enforce and refine regulations; businesses and manufacturers must innovate and maintain high standards; and the public should remain engaged and informed. This collective effort will ensure that the prepared dishes industry grows and contributes positively to public health and safety.

## 5 Limitations and future research

### 5.1 Limitations

It should be noted that, due to various limitations, this study was unable to establish a causal relationship between the characteristics of prepared dishes and public responses. Instead, the research was confined to objectively presenting the psychological perceptions of the public on specific health-related topics associated with prepared dishes.

Utilizing machine learning techniques, this research conducted sentiment analysis and topic clustering on comments from Chinese social media regarding prepared dishes. The findings indicate a generally negative sentiment among the Chinese public concerning the potential health risks associated with these dishes. For topic clustering, this study employed the BERTopic model, which demands considerable computational power. Thus, the corpus size used in the research is constrained. Moreover, given the strong correlation between food and public health, alongside cultural differences, it is anticipated that both the promotion of prepared dishes in China and the enhancement of public acceptance will undergo a protracted and dynamic process. Consequently, the conclusions of this study are confined to the objective presentation of current data, reflecting these limitations.

### 5.2 Future research

The large-scale promotion and popularization of prepared dishes in China are likely to be significant, making the accumulated experience from this study valuable for long-term future research. Due to constraints in resources and time, this study did not delve into fine-grained aspects such as population segmentation and geographical distribution, areas that merit further exploration. Additionally, to enhance the precision and efficiency of future research, serious consideration should be given to the selection of more advanced machine learning models. Particularly, with advancements in large-scale natural language processing models, an alternative avenue for expanding this research is presented, suggesting potential improvements in approaches.

## References

[1] Chen LA House L. Food lifestyle patterns among contemporary food shoppers. Int J Consum Stud. (2022) 46:944–63. doi: 10.1111/ijcs.12739

[2] Imtiyaz H Soni P Yukongdi V. Understanding Consumer’s purchase intention and consumption of convenience food in an emerging economy: role of marketing and commercial determinants. J Agric Food Res. (2022) 10:100399. doi: 10.1016/j.jafr.2022.100399

[3] Statista (2024). Convenience Food – Worldwide. Available at: https://www.statista.com/outlook/cmo/food/convenience-food/worldwide#revenue (Accessed February 26, 2024).

[4] iiMedia (2023). China’s pre-made food industry: Multiple policy benefits, comprehensive acceleration of China’s pre-made food industry. Available at: https://www.iimedia.cn/c1020/92136.html/ (Accessed February 26, 2024).

[5] Bhatt Y Jyothi Lakshmi AS. Effect of processing treatments on digestibility and physicochemical properties of ready-to-cook breakfast mixes. Food Proces Preserv. (2022) 46:e16324. doi: 10.1111/jfpp.16324

[6] Guo J Zhang M Law CL Luo Z. 3D printing technology for prepared dishes: printing characteristics, applications, challenges and prospects. Crit Rev Food Sci Nutr. (2023) 63:1–17. doi: 10.1080/10408398.2023.2238826, PMID: 37480290

[7] Tang T Zhang M Lim Law C Mujumdar AS. Novel strategies for controlling nitrite content in prepared dishes: current status, potential benefits, limitations and future challenges. Food Res Int. (2023) 170:112984. doi: 10.1016/j.foodres.2023.112984, PMID: 37316019

[8] Yu Q Zhang M Ju R Mujumdar AS Wang H. Advances in prepared dish processing using efficient physical fields: a review. Crit Rev Food Sci Nutr. (2022) 64:4031–45. doi: 10.1080/10408398.2022.2138260 36300891

[9] Huang M Zhang M Bhandari B. Recent development in the application of alternative sterilization technologies to prepared dishes: a review. Crit Rev Food Sci Nutr. (2019) 59:1188–96. doi: 10.1080/10408398.2017.1421140, PMID: 29359947

[10] Yadav B Spinelli AC Misra NN Tsui YY McMullen LM Roopesh MS. Effect of in-package atmospheric cold plasma discharge on microbial safety and quality of ready-to-eat ham in modified atmospheric packaging during storage. J Food Sci. (2020) 85:1203–12. doi: 10.1111/1750-3841.15072, PMID: 32118300

[11] Dinu M Tristan Asensi M Pagliai G Lotti S Martini D Colombini B. Consumption of ultra-processed foods is inversely associated with adherence to the Mediterranean diet: a cross-sectional study. Nutrients. (2022) 14:2073. doi: 10.3390/nu14102073, PMID: 35631214 PMC9147239

[12] Petimar J Grummon AH Simon D Block JP. Nutritional composition and purchasing patterns of supermarket prepared foods over time. Am J Prev Med. (2023) 64:213–20. doi: 10.1016/j.amepre.2022.08.021, PMID: 36280402 PMC9976399

[13] Namdar A Naghizadeh MM Zamani M Montazeri A. Exploring the relationship between health literacy and fast food consumption: a population-based study from southern Iran. BMC Public Health. (2021) 21:757. doi: 10.1186/s12889-021-10763-3, PMID: 33879106 PMC8056591

[14] Sun Y Zhang M Bhandari B Bai B. Nanoemulsion-based edible coatings loaded with fennel essential oil/cinnamaldehyde: characterization, antimicrobial property and advantages in pork meat patties application. Food Control. (2021) 127:108151. doi: 10.1016/j.foodcont.2021.108151

[15] Yi B Xu H. Research and Development status of prepared foods in China: a review. Appl Sci. (2023) 13:7998. doi: 10.3390/app13147998

[16] Wang J Gao Q Lou W. Development status and trends of the pre-prepared food industry in China. Modern Food Sci Technol. (2023) 39:99–103. doi: 10.13982/j.mfst.1673-9078.2023.2.0388

[17] Xiong Y Lin X Wen X Wang Y Liang W Xing T. The analysis of residents’ intention to consume pre-made dishes in China: a grounded theory. Food Secur. (2023) 12:3798. doi: 10.3390/foods12203798, PMID: 37893691 PMC10606755

[18] Liu J Li H Meng T Wu X. A consumer survey on cognition and purchase intention of pre-prepared food. Food Nut China. (2023):1–9. doi: 10.19870/j.cnki.11-3716/ts.20230315.001

[19] Zhang W Zheng J Li Y. Explaining Chinese consumers’ continuous consumption intention toward prepared dishes: the role of perceived risk and trust. Food Secur. (2023) 13:88. doi: 10.3390/foods13010088, PMID: 38201116 PMC10778665

[20] Zhang Q. Analysis of consumption trends and influencing mechanisms of prepared dishes based on SOR-SEM model. Manag Admin. (2023):1–12. doi: 10.16517/j.cnki.cn12-1034/f.20230712.005

[21] Zhong X. (2022). Prepared dishes must be not only popular but also tasty. China Consumer News. Available at: https://www.ccn.com.cn/Content/2022/02-23/0101520159.html (Accessed February 26, 2024).

[22] iiMedia (2023). Consumer awareness survey data on the safety of pre-prepared food in China. Available at: https://www.iimedia.cn/c1077/95893.html (Accessed February 26, 2024).

[23] Abbate S Centobelli P Cerchione R. The digital and sustainable transition of the Agri-food sector. Technol Forecast Soc Chang. (2023) 187:122222. doi: 10.1016/j.techfore.2022.122222

[24] Yoo R Kim S-Y Kim D-H Kim J Jeon YJ Park JHY. Exploring the nexus between food and veg*n lifestyle via text mining-based online community analytics. Food Qual Prefer. (2023) 104:104714. doi: 10.1016/j.foodqual.2022.104714

[25] Gefen D Larsen K. Controlling for lexical closeness in survey research: a demonstration on the technology acceptance model. JAIS. (2017) 18:727–57. doi: 10.17705/1jais.00469

[26] Podsakoff PM MacKenzie SB Podsakoff NP. Sources of method bias in social science research and recommendations on how to control it. Annu Rev Psychol. (2012) 63:539–69. doi: 10.1146/annurev-psych-120710-100452 21838546

[27] Elfenbein HA. Emotion in organizations: theory and research. Annu Rev Psychol. (2023) 74:489–517. doi: 10.1146/annurev-psych-032720-035940 36104000

[28] Oatley K Keltner D Jenkins JM. Understanding emotions, vol. xxvi. 2nd ed. Malden: Blackwell Publishing (2006). 508 p.

[29] Singh A Glińska-Neweś A. Modeling the public attitude towards organic foods: a big data and text mining approach. J Big Data. (2022) 9:2. doi: 10.1186/s40537-021-00551-6, PMID: 35013700 PMC8733915

[30] Tao D Yang P Feng H. Utilization of text mining as a big data analysis tool for food science and nutrition. Comp Rev Food Sci Food Safe. (2020) 19:875–94. doi: 10.1111/1541-4337.12540, PMID: 33325182

[31] Wang T Wang W Feng J Fan X Guo J Lei J. A novel user-generated content-driven and Kano model focused framework to explore the impact mechanism of continuance intention to use mobile APPs. Comput Hum Behav. (2024):108252. doi: 10.1016/j.chb.2024.108252

[32] Zhang C. (2023). With 4.74 billion users, what kind of social media is needed? Available at: https://www.huxiu.com/article/807827.html (Accessed February 26, 2024).

[33] Inwood O Zappavigna M. Conspiracy theories and white supremacy on YouTube: exploring affiliation and legitimation strategies in YouTube comments. Soc Media Soc. (2023) 9:205630512211504. doi: 10.1177/20563051221150410

[34] Trillo JR Herrera-Viedma E Morente-Molinera JA Cabrerizo FJ. A large scale group decision making system based on sentiment analysis cluster. Inform Fusion. (2023) 91:633–43. doi: 10.1016/j.inffus.2022.11.009

[35] Wu Z Zhang Y Chen Q Wang H. Attitude of Chinese public towards municipal solid waste sorting policy: a text mining study. Sci Total Environ. (2021) 756:142674. doi: 10.1016/j.scitotenv.2020.142674, PMID: 33071141

[36] Nazir S Khadim S Ali Asadullah M Syed N. Exploring the influence of artificial intelligence technology on consumer repurchase intention: the mediation and moderation approach. Technol Soc. (2023) 72:102190. doi: 10.1016/j.techsoc.2022.102190

[37] Martí P Serrano-Estrada L Nolasco-Cirugeda A. Social media data: challenges, opportunities and limitations in urban studies. Comput Environ Urban Syst. (2019) 74:161–74. doi: 10.1016/j.compenvurbsys.2018.11.001

[38] Shen Z. Mining sustainable fashion e-commerce: social media texts and consumer behaviors. Electron Commer Res. (2023) 23:949–71. doi: 10.1007/s10660-021-09498-5

[39] Carr J Decreton L Qin W Rojas B Rossochacki T Yang YW. Social media in product development. Food Qual Prefer. (2015) 40:354–64. doi: 10.1016/j.foodqual.2014.04.001

[40] Li C Niu Y Wang L. How to win the green market? Exploring the satisfaction and sentiment of Chinese consumers based on text mining. Comput Hum Behav. (2023) 148:107890. doi: 10.1016/j.chb.2023.107890

[41] Bilro RG Loureiro SMC Dos Santos JF. Masstige strategies on social media: the influence on sentiments and attitude toward the brand. Int J Consum Stud. (2022) 46:1113–26. doi: 10.1111/ijcs.12747

[42] Liu M Luo X Lu W-Z. Public perceptions of environmental, social, and governance (ESG) based on social media data: evidence from China. J Clean Prod. (2023) 387:135840. doi: 10.1016/j.jclepro.2022.135840

[43] Fatemi H Kao E Schillo RS Li W Du P Jian-Yun N. Using social media to analyze consumers’ attitude toward natural food products. BFJ. (2023) 125:3145–59. doi: 10.1108/BFJ-06-2022-0511

[44] Samoggia A Riedel B Ruggeri A. Social media exploration for understanding food product attributes perception: the case of coffee and health with twitter data. BFJ. (2020) 122:3815–35. doi: 10.1108/BFJ-03-2019-0172

[45] Chen Y Zhang Z. Exploring public perceptions on alternative meat in China from social media data using transfer learning method. Food Qual Prefer. (2022) 98:104530. doi: 10.1016/j.foodqual.2022.104530

[46] Zhang H Zhang D Wei Z Li Y Wu S Mao Z. Analysis of public opinion on food safety in greater China with big data and machine learning. Curr Res Food Sci. (2023) 6:100468. doi: 10.1016/j.crfs.2023.100468, PMID: 36891545 PMC9988419

[47] Zhou L Zhang C Liu F Qiu Z He Y. Application of deep learning in food: a review. Comp Rev Food Sci Food Safe. (2019) 18:1793–811. doi: 10.1111/1541-4337.12492 33336958

[48] Hamilton LM Lahne J. Fast and automated sensory analysis: using natural language processing for descriptive lexicon development. Food Qual Prefer. (2020) 83:103926. doi: 10.1016/j.foodqual.2020.103926

[49] Hirschberg J Manning CD. Advances in natural language processing. Science. (2015) 349:261–6. doi: 10.1126/science.aaa8685 26185244

[50] Mostafa MM. Mining and mapping halal food consumers: a geo-located twitter opinion polarity analysis. J Food Prod Mark. (2018) 24:858–79. doi: 10.1080/10454446.2017.1418695

[51] Couldry N Powell A. Big data from the bottom up. Big Data Soc. (2014) 1:205395171453927. doi: 10.1177/2053951714539277

[52] Zhong L Morrison AM Zheng C Li X. Destination image: a consumer-based, big data-enabled approach. TR. (2023) 78:1060–77. doi: 10.1108/TR-04-2022-0190

[53] Ghani NA Hamid S Targio Hashem IA Ahmed E. Social media big data analytics: a survey. Comput Hum Behav. (2019) 101:417–28. doi: 10.1016/j.chb.2018.08.039

[54] Lee I. Social media analytics for enterprises: typology, methods, and processes. Bus Horiz. (2018) 61:199–210. doi: 10.1016/j.bushor.2017.11.002

[55] Li X Xu M Zeng W Tse YK Chan HK. Exploring customer concerns on service quality under the COVID-19 crisis: a social media analytics study from the retail industry. J Retail Consum Serv. (2023) 70:103157. doi: 10.1016/j.jretconser.2022.103157

[56] Stieglitz S Mirbabaie M Ross B Neuberger C. Social media analytics – challenges in topic discovery, data collection, and data preparation. Int J Inf Manag. (2018) 39:156–68. doi: 10.1016/j.ijinfomgt.2017.12.002

[57] Cyberspace Administration of China (2016). Cybersecurity Law of the People’s republic of China. Available at: http://www.cac.gov.cn/2016-11/07/c_1119867116.htm (Accessed August 1, 2024).

[58] Glasze G Cattaruzza A Douzet F Dammann F Bertran M-G Bômont C. Contested Spatialities of digital sovereignty. Geopolitics. (2023) 28:919–58. doi: 10.1080/14650045.2022.2050070

[59] Su C Tang W. Data sovereignty and platform neutrality – a comparative study on TikTok’s data policy. Glob Media China. (2023) 8:57–71. doi: 10.1177/20594364231154340

[60] KAWO Guide to China Social Media Platforms (2023). Available at: https://share-eu1.hsforms.com/1CMfc4j7SQiWeNCE9GwdzmAfbvon?__hstc=14499944.39e4be92856ed27bbc8eead7b38fd437.1722682185678.1722682185678.1722682185678.1&__hssc=14499944.11.1722682185678&__hsfp=1989798536 (Accessed August 1, 2024).

[61] Mitchell R. (2018). Web scraping with Python: Collecting more data from the modern web. Sebastopol, CA, US: O’Reilly Media.

[62] Sharma S Gupta P. (2015). “The anatomy of web crawlers.” in *International conference on computing, Communication & Automation. Greater Noida, India*. IEEE pp. 849–853.

[63] Zhao B. Web scraping In: Schintler LA McNeely CL, editors. Encyclopedia of big data. Cham: Springer International Publishing (2017). 1–3.

[64] Kumar M Bindal A Gautam R Bhatia R. Keyword query based focused web crawler. Proc Comp Sci. (2018) 125:584–90. doi: 10.1016/j.procs.2017.12.075

[65] Jarmul K Lawson R. Python web scraping. Second ed. Birmingham: Packt Publishing (2017).

[66] Wang Q Zhang W Li J Mai F Ma Z. Effect of online review sentiment on product sales: the moderating role of review credibility perception. Comput Hum Behav. (2022) 133:107272. doi: 10.1016/j.chb.2022.107272

[67] Huang H Long R Chen H Sun K Li Q. Exploring public attention about green consumption on Sina Weibo: using text mining and deep learning. Sustain Prod Consump. (2022) 30:674–85. doi: 10.1016/j.spc.2021.12.017

[68] Salvatore C Biffignandi S Bianchi A. Social media and twitter data quality for new social indicators. Soc Indic Res. (2021) 156:601–30. doi: 10.1007/s11205-020-02296-w

[69] Ibrahim NF Wang X. Decoding the sentiment dynamics of online retailing customers: time series analysis of social media. Comput Hum Behav. (2019) 96:32–45. doi: 10.1016/j.chb.2019.02.004

[70] Barde BV Bainwad AM. (2017). “An overview of topic modeling methods and tools.” in *2017 international conference on intelligent computing and control systems (ICICCS) Madurai*. IEEE. pp. 745–750.

[71] Blei DM Ng AY Jordan MI. Latent Dirichlet allocation In: Dietterich TG Becker S Ghahramani Z, editors. Advances in neural information processing systems 14: Cambridge, MA, US: The MIT Press (2002). 601–608.

[72] Hofmann T. (2013). Probabilistic Latent Semantic Analysis. doi: 10.48550/ARXIV.1301.6705 (Accessed February 26, 2024)

[73] Grootendorst M. (2024). BERTopic: Neural topic modeling with a class-based TF-IDF procedure. (Accessed February 26, 2024).

[74] Huang H Long R Chen H Sun K Sun Q Li Q. Why don’t more people engage in green practices in China? A policy-oriented approach to promoting green transformation in five consumption areas. Environ Impact Assess Rev. (2023) 101:107099. doi: 10.1016/j.eiar.2023.107099

[75] Oh YK Yi J Kim J. (2023). What enhances or worsens the user-generated metaverse experience? An application of BERTopic to Roblox user eWOM. INTR.

[76] Ebeling R Nobre J Becker K. A multi-dimensional framework to analyze group behavior based on political polarization. Expert Syst Appl. (2023) 233:120768. doi: 10.1016/j.eswa.2023.120768

[77] Uncovska M Freitag B Meister S Fehring L. Rating analysis and BERTopic modeling of consumer versus regulated mHealth app reviews in Germany. NPJ Digit Med. (2023) 6:115. doi: 10.1038/s41746-023-00862-3, PMID: 37344556 PMC10285024

[78] Shen C Wang A Fang Z Zhang Q. Trend mining of product requirements from online reviews. Chin J Manag Sci. (2021) 29:211–20. doi: 10.16381/j.cnki.issn1003-207x.2018.1508

[79] Geary RC Kendall MG. Rank correlation methods. Econ J. (1949) 59:575. doi: 10.2307/2226580

[80] Mann HB. Nonparametric tests against trend. Econometrica. (1945) 13:245. doi: 10.2307/1907187

[81] Danner H Menapace L. Using online comments to explore consumer beliefs regarding organic food in German-speaking countries and the United States. Food Qual Prefer. (2020) 83:103912. doi: 10.1016/j.foodqual.2020.103912

[82] DT Business Observer (2024). Only 9.1% of people are against pre-prepared meals. Available at: https://inf.news/en/news/dc5f84ebfd39a294c86385bade626b4b.html (Accessed July 31, 2024).

[83] Wang Z Jin Y Liu Y Li D Zhang B. Comparing social media data and survey data in assessing the attractiveness of Beijing Olympic Forest Park. Sustain For. (2018) 10:382. doi: 10.3390/su10020382

[84] Zeng G Chen Z Zhong S. “We Chinese just want meat!” an analysis of Chinese netizens’ reactions to vegetarian advocacy. Food Qual Prefer. (2024) 115:105128. doi: 10.1016/j.foodqual.2024.105128

[85] Zhang L Zhou F Zhou C. The bottleneck restriction and path selection of China’s prepared meals industry from the perspective of high-quality development. Food Ferment Indust. (2024) 50:381–8. doi: 10.13995/j.cnki.11-1802/ts.037771

[86] Jing L Liao Q Luo W Jian Q Zhuang Y. Research on the development status and countermeasures of prepared dishes industry. China Food Safety Magazine. (2024) 6:187–9. doi: 10.16043/j.cnki.cfs.2024.06.032

